# Complementary feeding in preterm infants: a position paper by Italian neonatal, paediatric and paediatric gastroenterology joint societies

**DOI:** 10.1186/s13052-022-01275-w

**Published:** 2022-08-05

**Authors:** Maria Elisabetta Baldassarre, Raffaella Panza, Francesco Cresi, Guglielmo Salvatori, Luigi Corvaglia, Arianna Aceti, Maria Lorella Giannì, Nadia Liotto, Laura Ilardi, Nicola Laforgia, Luca Maggio, Paolo Lionetti, Carlo Agostoni, Luigi Orfeo, Antonio Di Mauro, Annamaria Staiano, Fabio Mosca, On behalf of Italian Society of Paediatrics (SIP), Italian Society of Neonatology (SIN) and Italian Society of Paediatric Gastroenterology, Hepatology and Nutrition (SIGENP)

**Affiliations:** 1 Department of Biomedical Science and Human Oncology, Section of Neonatology and Neonatal Intensive Care Unit, “Aldo Moro” University of Bari, Policlinico Hospital - Piazza Giulio Cesare n. 11, 70124 Bari, Italy; 2 Neonatology and Neonatal Intensive Care Unit, “A. Perrino” Hospital, Brindisi, Italy; 3 Neonatology and Neonatal Intensive Care Unit, Department of Public Health and Paediatrics, University of Turin, Torino, Italy; 4 Neonatal Intensive Care Unit and Human Milk Bank, Department of Neonatology, Bambino Gesù Children’s Hospital, IRCCS, Rome, Italy; 5 Department of Medical and Surgical Sciences - University of Bologna, Neonatal Intensive Care Unit - IRCCS AOUBO, Bologna, Italy; 6 Fondazione IRCCS Ca’ Granda Ospedale Maggiore Policlinico, Neonatal Intensive Care Unit, 20122 Milan, Italy; 7 Department of Clinical Sciences and Community Health, University of Milan, 20122 Milan, Italy; 8 Neonatology and Neonatal Intensive Care Unit ASST Grande Ospedale Metropolitano Niguarda, Milan, Italy; 9 Department of Interdisciplinary Medicine - Section of Neonatology and Neonatal Intensive Care Unit, “Aldo Moro” University of Bari, 70124 Bari, Italy; 10 UOC Neonatology and Neonatal Intensive Care Unit, AO San Camillo Forlanini, Rome, Italy; 11 Gastroenterology Unit, NEUROFARBA Department, University of Florence, Meyer Children’s Hospital, Florence, Italy; 12 Fondazione IRCCS Ca’ Granda Ospedale Maggiore Policlinico, Paediatric Intermediate Care Unit, Milan, Italy; 13 Neonatal Intensive Care Unit, “San Giovanni Calibita Fatebenefratelli” Hospital, Isola Tiberina, Rome, Italy; 14 Paediatric Primary Care, National Paediatric Health Care System, Via Conversa 12, Margherita di Savoia, BT Italy; 15 Department of Translational Medical Science, Section of Paediatrics, University of Naples “Federico II”, Naples, Italy

**Keywords:** Nutrition, Complementary feeding, Weaning [Mesh], Breastfeeding [Mesh], Breast milk [Mesh], Fortification, Infant, Premature [Mesh], Preterm, Births [Mesh]

## Abstract

Nutrition in the first 1000 days of life is essential to ensure appropriate growth rates, prevent adverse short- and long-term outcomes, and allow physiologic neurocognitive development. Appropriate management of early nutritional needs is particularly crucial for preterm infants. Although the impact of early nutrition on health outcomes in preterm infants is well established, evidence-based recommendations on complementary feeding for preterm neonates and especially extremely low birth weight and extremely low gestational age neonates are still lacking. In the present position paper we performed a narrative review to summarize current evidence regarding complementary feeding in preterm neonates and draw recommendation shared by joint societies (SIP, SIN and SIGENP) for paediatricians, healthcare providers and families with the final aim to reduce the variability of attitude and timing among professionals.

## Main text

### Introduction

Nutrition in the first 1000 days of life can help ensure appropriate growth rates and prevent adverse short- and long-term outcomes in infants [1]. Early nutrition is also essential for physiologic neurocognitive development [2–4]. Appropriate management of early nutritional needs is particularly crucial for preterm infants, a vulnerable population that features specific nutritional requirements which differ from those of term neonates [5]. Prematurity (defined as birth before 37 weeks gestational age [GA]) still affects 7–11% of live births worldwide every year [6, 7] and it represents a significant cause of mortality and morbidity not only in the first years of life, but also later in life. Premature infants frequently develop postnatal growth retardation [8] and feature an altered body composition [9, 10], with reduced fat free mass and increased adiposity [9–12].

Although the impact of early nutrition on health outcomes in preterm infants is well established, evidence-based recommendations on complementary feeding (CF) for preterm neonates and especially for extremely low birth weight (ELBW) and extremely low gestational age neonates (ELGAN) are still lacking. CF (also called weaning) is defined by the World Health Organization as “the process starting when breast milk alone is no longer sufficient to meet the nutritional requirements of infants” so that “other foods and liquids are needed, along with breast milk” [13]. It plays a pivotal role in infantile nutrition and neurodevelopment, and represents a delicate period in which either nutritional deficits or overfeeding may be exacerbated.

What is known is that guidelines for CF in term infants [14, 15] are not appropriate for preterm neonates, hence the urgency for specific recommendation for premature babies [3, 16, 17].

The objective of this position paper is to summarize current evidence regarding CF in preterm neonates and provide recommendation shared by joint societies (Italian Paediatric Society - SIP, Italian Society of Neonatology - SIN, and Italian Society of Paediatric Gastroenterology, Hepatology and Nutrition - SIGENP) for paediatricians, healthcare providers and families with the final aim to reduce the variability of attitude [18] and timing [19] among professionals.

### Who is it for?

- Paediatricians and healthcare providers involved in the care of preterm neonates and preterm infants
- Parents and carers of preterm neonates and preterm infants

## Materials and methods

A recommendation development committee was created including neonatologists, paediatricians and nutrition experts. Parent representatives were also surveyed at multiple points during the process.

The target population was determined to be preterm neonates (GA < 37 weeks) and committee members were assigned topics based on expertise.

For each topic, screening was performed according to Preferred Reporting Items for Systematic Review and Meta-Analysis (PRISMA) guidelines [20]. The following keywords and Mesh terms were employed: complementary food; complementary feeding; weaning; introduction; timing; preterm newborn; premature; preterm infants; health outcome; development; adiposity rebound; paediatric obesity; body mass index; nutrition; post-discharge formula; macronutrients; oral dysfunction; allergy; “Weaning”[Mesh]; “Infant, newborn”[Mesh]; “Diet, vegetarian”[Mesh]; “Diet, vegan” [Mesh]. Proper Boolean operators “AND” and “OR” were also included to be as comprehensive as possible. Search limits were set for studies published up to 31st August 2021 in English language. Eligible studies were retrieved using the PubMed, Embase, Cochrane Library and Web of Science databases. Additional studies were identified from conference proceedings, trial registries and the reference lists of the selected papers. As a result, 62 manuscripts were selected for this position paper, including 8 systematic reviews [3, 18, 21–26], 8 narrative reviews [27–34], 27 observational studies [19, 35–60], 4 controlled trials [61–64], 1 case report [65], 3 commentaries [66–68], 1 operational protocol [69], 3 reports [70–72], 1 consensus [73], 2 recommendations [74, 75], 2 guidelines [76, 77] and 3 nutritional reference values [78–80]. One or more recommendations/statements were drafted for each topic. Grading of Recommendations, Assessment, Development and Evaluation (GRADE) approach [81] was used to assess the quality of evidence (i.e., high, moderate, low or very low) and to define the strength of the recommendations (i.e., weak or strong) according to potential desirable and undesirable consequences of the recommendation. Final recommendations and statements were reviewed by experts and future guideline users to ensure feasibility. Based on available data, recommendations and statements were proposed, discussed and rephrased until a consensus of 90% or more was reached.

### When should complementary feeding be started?

The timing for introduction of solid foods in preterm infants is still a matter of debate. Different timeframes were suggested in the past such as 3–6 months of postnatal age (PA) [70, 71], 5–8 months of PA [72, 76] or more recently from 3 months of corrected age (CA) [18].

The majority of data on CF in preterm infants were derived from observational studies, thus reducing the robustness of the recommendations. Only few randomized controlled trials have assessed differences between timings of CF introduction (Table 1). Marriott et al. divided 68 preterm infants in two groups: “preterm weaning strategy (PWS)” group or control group. The PWS group was weaned at 13 weeks of age and at least 3.5 kg body weight compared to 17 weeks of age and at least 5 kg. The PWS group also received advice regarding quality of foods, encouraging the consumption of high-energy and high-protein foods, and a mixture of dried cereals and home-prepared foods with preterm infant formula. Their results show that the PWS featured greater length at 12 months of age, with no differences in weight or head circumference, compared to the control group [61]. A prospective cohort study by Spiegler et al. [35] showed in a regression analysis that length and weight of VLBW infants at 24 months were positively influenced by early introduction of CF: VLBW infants at 24 months of age were on average ~ 0.4 cm taller and 100 g heavier for each month of earlier introduction of CF. Also Rodriguez et al. found a beneficial effect of weaning before 4 months of CA with higher weight gain at 18–24 months of CA in very preterm infants [46].

**Table 1:** Main features of RCTs and observational studies assessing timing for CF introduction in preterm infants

Author, Year	Study Design	Sample Size	Results
Baldassarre, 2018 [19]	Observational study	Survey of CF practices among 347 Italian primary care paediatricians	Wide heterogeneity in CF timing (based on age or neurodevelopmental skills or body weight), quality, and prescription of vitamin D and iron supplements.
Marriott, 2003 [61]	RCT	﻿ RCT comparing PWS vs. conventional CF management in 68 preterm infants randomised to either the PWS group (*n* = 37) or control group (*n* = 31)	Infants in the PWS group showed higher length scores and length growth velocity, and higher intake of energy, carbohydrate, protein, and iron during follow up.
Spiegler, 2015 [35]	Observational study	Longitudinal analysis of introduction of CF in 981 German VLBW infants, risk factors for early introduction of CF, and relationship between age at CF start and growth at 2 years of age	﻿Average introduction of CF was 3.5 months CA. Lower GA correlated with earlier introduction of vegetables and meat. Age at introduction of CF was influenced by IUGR, GA at birth, maternal education and a developmental delay perceived by parents. No negative effect of early introduction of CF on length and weight at 2 years of age.
Rodriguez, 2018 [46]	Observational pilot study	Cross-sectional study assessing the relationship between feeding practices and weight gain at 18–24 months CA in 36 toddlers born < 32 weeks’ GA	Forty-one % infants received CF before four months CA. A greater weight gain was observed in infants on early CF.
Gupta, 2017 [62]	RCT	RCT comparing CF starting at 4 vs. 6 months CA in 373 Indian preterm infants born < 34 weeks’ GA (*n* = 184 CF at 4 months CA vs. *n* = 189 CF at 6 months CA)	No difference was found in weight-for-age z score at 12 months CA between groups, but a higher hospitalization rate was documented in the 4 month CF group.
Morgan, 2004 [21]	Pooled RCTs results	Pooled results from 5 RCTs assessing early (< 12 weeks) vs. late (> 12 weeks) introduction of CF in 1694 term and preterm infants	Preterm infants weaned before 12 weeks featured slower increase in weight, length, and head circumference at 12 weeks − 18 months; by 18 months, there were no significant differences in size between the two groups.
Zielinska, 2019 [54]	Observational study	﻿﻿Cross-sectional study investigating *n* = 5815 parents of infants aged 1–3 years from Poland (*n* = 4065) and Austria (*n* = 1750) using a single online questionnaire	Cross-sectional study assessing risk factors for early CF in Poland and Austria.Preterm birth was a significant risk factors for early CF, together with lower maternal age and educational level, absence of breastfeeding and formula feeding after hospital discharge.
Cleary, 2020 [55]	Observational study	﻿Prospective longitudinal study on 150 ﻿infants (preterm *n* = 85; term *n* = 65)	Structured interviews on infant feeding practices, growth and medical status in term and preterm infants. Preterm infants received CF earlier than term infants; lower maternal education and male gender were significant risk factors for early CF.
Fanaro, 2007 [56]	Observational study	Survey of CF practices in an Italian region on *n* = 156 infants	Significant variation in timing and inappropriate equality of CF (low energy, protein, iron and zinc content). Maternal age significantly influenced the weaning schedule.
Norris, 2002 [57]	Observational study	﻿Two-hundred and fifty-three preterm infants (139 male, 114 female) assessed by structured interviews in the UK	Nearly half of the sample received early CF. Formula-fed infants (mean age at CF from term 10.2 ± 0.47 weeks) were weaned significantly earlier than both human milk-fed (11.9 ± 0.49 weeks; *p* < 0.05) and combined milk-fed (11.9 ± 0.25 weeks; *p* < 0.005) infants.
Braid, 2015 [58]	Observational study	Multivariate logistic regression on 7650 infants (term vs. preterm)	Higher odds of early CF in ELGAN. Lower GA was associated with higher odds of early CF.
Giannì, 2018 [59]	Observational study	﻿Assessment of CF practices in a cohort of 64 Italian late preterm infants	Late preterm infants started CF at almost six months of age receiving first solid foods with low energy and protein content.
Menezes, 2018 [60]	Observational study	﻿Cross-sectional study on 38 preterm infants ﻿to investigate difficulties in CF in premature infants	Nearly 75% of preterm infants experienced at least one defensive behaviour at mealtime (e.g., refusal to open their mouth, food selectivity, and feeding refusal).
Crapnell, 2013 [36]	Observational study	﻿Assessment of early medical and family factors associated with later CF in 136 preterm infants (≤30 weeks’ GA)	Nearly a quarter of infants experienced feeding problems at 2 years. Early hypotonia and lower socio-economic status were documented as risk factors for delayed CF.

*CA* corrected age, *CF* complementary feeding, *ELGAN* extremely low gestational age neonate, *GA* gestational age, *IUGR* intrauterine growth restriction, *RCT* randomized controlled trial.

Conversely, an RCT conducted in India to compare two different timings of CF (4 vs. 6 months CA) in ex preterm neonates with GA < 34 weeks revealed that weight-for-age z score at 12 months CA did not differ between groups, but the 4-month CA group experienced a higher rate of hospital admission primarily due to infectious disease [62]. Hence authors recommend to delay CF until 6 months CA in this population, however generalisability of their findings is uncertain due to the important differences between low and high income countries, including higher mortality rate, environmental conditions, and predominantly vegetarian dietary regimens [68].

Similarly, a pooled analysis of prospective studies by Morgan et al. [21] showed no effects on height and weight at 24 months of age and health outcomes up to 18 months.

What is known is that preterm infants are usually weaned early (before 4 months of age) compared to their term counterpart [3, 54–57]. Moreover, the first solid food is often nutritionally inadequate, with a low energy and protein content [56], and wide variability in weaning practices and vitamin and iron supplementations [19]. The entity of prematurity influences greatly the timing of weaning: preterm infants born at 22–32 weeks GA show a 9.90 odds of receiving CF before 4 months of age, compared to term peers [58].

However, also late preterm infants are often weaned early, at a mean postnatal age of 5.7 months and a mean CA of 4.6 months [59].

The early introduction of solid foods in preterm infants has been linked to a higher risk of rapid weight gain [46], allergy and anaemia whilst a delayed weaning (after 7–10 months of PA) may increase the risk of avoidance feeding behaviour [18].

It is noticeable not only that preterm infants are introduced early to CF, but also that the attitude of primary care paediatricians is widely variable in terms of timing of introduction and type of suggested foods [19].

This is partly due to the lack of specific guidelines on CF introduction for preterm infants [22]. The COMA report in 1994 suggested weaning preterm infants with a body weight of at least 5 kg, provided they had acquired a few specific developmental milestones [72]. However, these suggestions may lead to a significant delay in some populations of preterm infants (e.g., ELGAN or ELBW) which would reach such criteria well beyond the timeframe (4–6 months of age) recommended by the ESPGHAN to start CF in term infants [14]. Preterm infants starting CF often show defensive behaviours at mealtime, such as refusal to open the mouth, food selectivity and feeding refusal [60]. More recently, it has been recommended that CF in preterm infants should be started between 5 and 8 months of chronological age [76] when neurodevelopmental skills (e.g. good control of the neck, disappearance of the protrusion reflex of the tongue, the reduction of reflexive suck in favour of lateral tongue movements, and the gradual appearance of lip seal) and readiness to explore new textures and flavours should have been reached by the vast majority of ex preemies. Since an adequate motor development is a pivotal requirement for starting CF, it has also been advised to consider CA in the assessment of the optimal timing for weaning preterm infants. In this respect the limit of 3 months CA has been set to ensure the acquisition of developmental skills which allow the consumption of solid foods. Importantly, CA would be a unifying criterion for the heterogeneous population of preterm infants, since it is applicable to babies of all gestational ages, from the lowest to the highest [3].

Although critical, neurodevelopmental readiness is not the only aspect to take into consideration. Difficult transition to complementary food may also be related to comorbidities, or even behavioural issues, which should be carefully assessed with the aid of a multidisciplinary team. Indeed, the multiple and unpleasant procedures undergone during hospital admission (e.g., orogastric/nasogastric tube feeding, suctioning, intubation) may lead to a negative attitude towards CF. Furthermore, parental emotional factors should not be underestimated, particularly in growth-restricted infants, whose growth rate is often concerning for parents [36, 60].

Currently, there is insufficient evidence to draw final conclusions regarding a specific timing for starting CF in preterm infants, due to their extreme variability in achieving neurodevelopmental and oral skills. Hence, we suggest an individualized approach based on the accurate evaluation of the infant development and attitude towards semi-solid foods, employing corrected or postnatal age as an indicative reference rather than a mandatory schedule.

*Recommendations/Statements*

- CF in preterm infants should be started between 5 and 8 months of chronological age.
- Consider also the limit of 3 months CA to ensure the acquisition of developmental skills which allow the consumption of solid foods.

*Certainty of evidence: Moderate.*

*Grade of recommendation: Strong.*

### Are there specific recommendations for preterm infants with oral dysfunction or comorbidities?

Oral dysfunction is not uncommon among infants born preterm, due to the higher occurrence of comorbidities (e.g., bronchopulmonary dysplasia) or neurodevelopmental impairment [27, 37]. Reportedly, over 15% of preterm infants require enteral tube feeding upon discharge [38]. Lower gestational ages at birth (below 30 weeks) and neonatal surgery have been described as risk factors for oro-motor feeding problems at 12 months’ CA [39]. This sub-group of infants often features greater defensive behaviours at mealtime when starting CF, e.g. refusal to open the mouth, feeding refusal and food selectivity [60].

However, guidelines regarding CF for preterm infants with oral dysfunction or major comorbidities are still lacking, hence the nutritional strategy for these infants should be tailored and revised regularly (Table 2). Seemingly, a greater amount of food is consumed by preterm infants using a spoon-assisted mode of feeding [63], probably due to the decreased gag reflex elicited by the introduction of food with higher texture [28]. CF may be started at 3–4 months of corrected GA, encouraging the consumption of thicker foods which may be swallowed more easily.

**Table 2:** Main features of trials and observational studies assessing preterm infants with oral dysfunction or comorbidities

Author, Year	Study Design	Sample Size	Results
Menezes, 2018 [60]	Observational study	﻿Cross-sectional study on 38 preterm infants ﻿to investigate difficulties in CF in premature infants	Nearly 75% of preterm infants experienced at least one defensive behaviour at mealtime (e.g., refusal to open their mouth, food selectivity, and feeding refusal).
Pahsini, 2018 [37]	Observational study	﻿Evaluation of prematurity rate among 711 tube dependent children from the program based on the “Graz Model of tube weaning”	According to ICD-10 classification, 378 children (53.2%) were born prematurely, with 103 infants < 29 weeks’ GA and 275 between 29 and 36 + 6 weeks’ GA.
Kamitsuka, 2017 [38]	Observational study	Assessment of the impact of an oral feeding protocol (OFP) on the number of infants requiring home tube feeds: the study included 129 infants before the protocol implementation and 141 infants afterwards	﻿After introducing the OFP, oral feedings were started earlier, full oral feedings were achieved sooner, and the incidence of home tube feeds at discharge was reduced.
Sanchez, 2016 [39]	Observational study	Evaluation of oro-motor feeding at 12 months’ CA in 90 infants born before 30 weeks’ GA vs. 137 term-born peers	Preterm infants featured greater odds of oro-motor feeding problems at 12 months’ CA. Neonatal surgery was documented as risk factor for feeding difficulties.
Malhotra, 1999 [63]	Cross-over controlled trial	Assessment of bottle, cup and a traditional feeding device (‘paladai’) in 100 infants (*n* = 66 term AGA infants, *n* = 20 term SGA infants, and *n* = 14 preterm infants)	﻿Infants took the maximum volume in the least time and kept quiet the longest with the paladai. Spilling was the highest with the cup, especially in preterm infants.

*AGA* appropriate for gestational age, *CA* corrected age, *CF* complementary feeding, *GA* gestational age, *OFP* oral feeding protocol, *SGA* small for gestational age.

Importantly, preterm infants with oral dysfunctions or comorbidities require a multidisciplinary follow up encompassing nutrition experts, speech therapists, and a behavioural psychologist [28, 29, 69]: oro-motor stimulation should be started early for infants on prolonged tube feeds. Infants with gastrointestinal issues should also be followed up by a paediatric gastroenterologist. Ex preemies with bronchopulmonary dysplasia should be weaned with low salt, limited volume, and high energy diets; these infants usually better tolerate foods given by spoon since they may suffer from mild hypoxic spells when suckling liquids.

Complete foods based on amino-acid mixtures concentrate in small volumes a high macronutrients content: they may be an option to meet the high nutritional requirements of infants with comorbidities or of those infants unable to ingest large quantities of food [3].

*Recommendations/Statements*

- Preterm infants with oral dysfunctions or comorbidities may require a multidisciplinary assessment to evaluate when and how CF should be started.

*Certainty of evidence: Low.*

*Grade of recommendation: Weak.*

### Which type of food should be recommended?

When it comes to solid foods for preterm infants, two critical aspects should be taken in consideration. Firstly, if the acceptance and consumption of semi-solid food is still inadequate, attention should be paid to the intake of micronutrients. In this respect, supplementation with iron and multivitamin products are helpful to ensure the correct supply of micronutrients. Secondly, if catch-up growth has not been reached by the time of weaning, a high protein and energy intake should be promoted by means of the correct formula or specific foods to propose. The choice of the right formula milk (i.e., post-discharge or standard formula) is also dependent on the milk tolerance of the infant, since less mature preterm infants may have immature feeding skills but higher energy requirements [3, 30].

Importantly, several figures are involved in the process of weaning a preterm infant: families, primary care paediatricians and nutrition experts. Each figure plays an important role. The family is pivotal since it represents the main support for the babies and their parents. According to a recent systematic review, nutrition education for families may decrease the risk of undernutrition in term infants [23], hence we may speculate that the same could occur with ex-preemies. The primary care paediatrician should carefully evaluate growth patterns and ensure adequate adherence to prescriptions and nutritional advice. Lastly, the nutritional expert should guide all the weaning process by carefully evaluating the infant nutritional needs and neurodevelopmental and oral skills, in order to provide tailored recommendations.

More specific recommendations for preterm infants regarding type of foods to choose, sequence and speed of introduction are lacking, hence guidelines for term infants currently remain the gold standard [14]. Importantly, the beginning of CF is associated with significant changes in both macronutrients and micronutrients intake, with the risk of nutritional imbalances. The energy requirement differs according to the degree of prematurity. Embleton et al. [40] showed that preterm infants often fail to meet their dietary intake (energy 102 kcal/kg/day; protein 3.0 g/kg/day) since the first days of life and that such deficits are not recovered by the time of discharge.

Recently, Salvatori et al. [24] suggested intake of macronutrients for preterm infants taking into account recommendations conceived by The Italian Society of Human Nutrition with LARNs (Reference intake Levels of Nutrients and energy for the Italian population) of 2014 [78], the Dietary Reference Values for nutrients of European Food Safety Authority (EFSA) of 2017 [79] and the Nutrient Reference Values for Australia and New Zealand Including Recommended Dietary Intakes of 2017 [80] (Table 3).

**Table 3:** Macronutrients adequate intake for infants

Macronutrient	Age	AI (Adequate Intake)
Water	6–12 months	800–1000 ml/day
Proteins	6–12 months	1.6 g/kg/day
Carbohydrates	7–12 months	95 g/day
Total Lipids	7–12 months	30 g/day
n-6 PUFA	7–12 months	4.6 g/day
n-3 PUFA	7–12 months	0.5 g/day

*PUFA* poli-unsaturated fatty acids.

As for micronutrients, iron supply is a matter of concern due to its essential role for brain development. Iron supplementation is recommended for preterm infants until at least 6–12 months of age [66]. However, from 6 months of age the supplementation alone would not be sufficient to provide the adequate amount of iron, hence the consumption of foods rich in iron (e.g., meat, iron-fortified cereals, fish) should be encouraged.

*Recommendations/Statements*

- Recommendations for preterm infants regarding type of foods to choose, sequence and speed of introduction may be considered the same as for term infants currently.
- Consider starting CF encompassing sources of carbohydrates, proteins and vegetable fats (extra-virgin olive oil) and paying special attention to the intake of micronutrients (e.g., iron and vitamins).

*Certainty of evidence: Low.*

*Grade of recommendation: Weak.*

### Is there a link between early CF and obesity?

Extrauterine growth retardation is very frequent in preterm infants that usually weigh significantly less than expected at hospital discharge and often remain small throughout infancy and childhood. However, an excessive protein supply in the first stages of life and the early introduction of CF have been linked to increased concentrations of insulin and insulin-like growth factor-1 (IGF-1), which in turn cause higher weight gain and body fat deposition leading to an increased risk of obesity. Singhal et al. demonstrated that ex-preterm patients aged 13–16 years featured higher fasting 32–33 split proinsulin concentrations if fed with a nutrient-enriched diet in early childhood (mean 7.2 pmol/l, 95% CI 6.4–8.1 vs 5.9 pmol/l 95% CI 5.2–6.4; *p* = 0.01). Fasting 32–33 split proinsulin levels were also associated with greater weight gain in the first two weeks of life, suggesting that early relative undernutrition in children born preterm may have beneficial effects on insulin resistance [64].

Hence, there is still uncertainty whether the early introduction of CF is more beneficial in short-term weight gain or, in contrast, it is more detrimental due to the long-term risk of obesity and metabolic syndrome [77].

A few studies explored the influence of early weaning on body mass index (BMI) in preterm infants (Table 4). Gupta et al. did not find any significant difference of BMI index z score at 12 months according to timing of CF [62], whereas Sun et al. showed that early CF introduction was negatively associated to BMI at 12 months of age [41]. In contrast, Morgan et al. showed that preterm infants weaned before 3 months CA featured a greater gain in the subscapular skinfold thickness between 3 and 9 months CA [21].

**Table 4:** Main features of RCTs and observational studies assessing the relationship between CF introduction in infants and later onset of obesity

Author, Year	Study Design	Sample size	Results
Singhal, 2003 [64]	RCT	Measurement of fasting 32–33 split proinsulin concentration in adolescent participants born preterm and randomised to receive a nutrient-enriched or lower-nutrient diet (*n* = 216) vs. a control group born at term (*n* = 61)	Relative undernutrition early in life in premature infants may have beneficial effects on insulin resistance.
Sun, 2016 [41]	Observational study	Cross-sectional, population-based study on 3153 Australian infants	Introduction of CF at 5–6 months, compared with either early or delayed introduction, is associated with decreased odds of above normal BMI.
Baldassarre, 2020 [42]	Observational study	Prospective, population-based longitudinal study on 100 preterm infants	Half of preterm neonates experienced early adiposity rebound and featured significantly higher BMI at seven years compared to children with timely adiposity rebound (17.2 ± 2.7 vs. 15.6 ± 2.05, *p* = 0.021).
Gupta, 2017 [62]	RCT	RCT comparing CF starting at 4 vs. 6 months CA in 373 Indian preterm infants born < 34 weeks’ GA (*n* = 184 CF at 4 months CA vs. *n* = 189 CF at 6 months CA)	No difference was found in weight-for-age z score at 12 months CA between groups, but a higher hospitalization rate was documented in the 4 month CF group.
Morgan, 2004 [21]	Pooled RCTs results	Pooled results from 5 RCTs assessing early (< 12 weeks) vs. late (> 12 weeks) introduction of CF in 1694 term and preterm infants	Preterm infants weaned before 12 weeks featured slower increase in weight, length, and head circumference at 12 weeks − 18 months; by 18 months, there were no significant differences in size between the two groups.
Baldassarre, 2017 [43]	Observational study	Survey exploring the influence of neonatal features on the onset of non-communicable diseases: *n* = 6379 questionnaires were assessed	Preterm birth was not associated with the onset of asthma and allergy, celiac disease or diabetes, and acted as a protective factor in the development of obesity.
Kaul, 2019 [44]	Observational study	Results from *n* = ﻿81,226 children ﻿with the aim to assess the association between gestational diabetes, being LGA at birth and breast feeding with overweight/obesity in early childhood	LGA is a strong risk factor for being overweight/obese in early childhood, especially in babies born to diabetic mothers. Breast feeding was a protective factor against overweight/obesity in childhood in the majority of children, except for LGA children of diabetic mothers.
Kapral, 2018 [45]	Observational study	Analysis of *n* = 10,186 term or preterm children in the Early Childhood Longitudinal Study-Kindergarten Cohort 2011 to assess relationships between BW and later obesity in childhood	High BW term and LGA preterm children had increased adjusted odds of obesity in childhood.
Brion, 2020 [47]	Observational study	Assessment of BMI, weight-for-length and head growth in 208 AGA infants born at 23–28 + 6 weeks’ GA	Infants started on ready-made CF ≤26 weeks CA had the highest BMI and weight-for-length at 12 months. Head growth from discharge to 12 months was the highest in infants either discharged on breastmilk or receiving home-made CF at ≤26 weeks' CA.
Fenton, 2021 [48]	Observational study	To examine the prevalence and risk factors for childhood overweight and obesity at 3-year CA in 911 preterm babies (BW < 1500 g or GA < 29 weeks)	Small size at birth or at 36 weeks’ GA in ELGAN is not associated with increased risk of early childhood overweight or obesity.

*AGA* appropriate for gestational age, *BMI* body mass index, *BW* birth weight, *CA* corrected age, *CF* complementary feeding, *ELGAN* extremely low gestational age neonates, *GA* gestational age, *LGA* large for gestational age, *RCT* randomized controlled trial

A recent study showed that half of preterm infants featured an early adiposity rebound (≤ 4 years of age) irrespective of timing of CF introduction [42], hence authors concluded that premature birth can be regarded as an independent risk factor for obesity and other non-communicable diseases later in life [25, 43]. The risk of being overweight or obese in early childhood is higher for small for gestational age (SGA) [31] and large for gestational age (LGA) [44, 45] neonates. An observational cohort study concluded that starting CF before 26 weeks of CA is associated with a higher BMI at 12 months of age in preterm infants [47]. Nonetheless, a recent systematic review regarding the link between the timing of CF in preterm infants and the incidence of overweight could not draw final conclusions due to the shortage of randomized controlled trials [26] and recent findings from a multicentre retrospective cohort study on 911 preterm infants demonstrated no associations between overweight or obesity at 3 years of age and risk factors such as extremely preterm infants being SGA or experiencing extrauterine growth retardation (EUGR) [48]. We could speculate that these contrasting findings might be due to either heterogeneity of study designs or different energy intakes between the periods of assessment.

*Recommendations/Statements*

- Timing of CF start in preterm infants is unlikely to influence the incidence of overweight and obesity in childhood and adulthood.
- The start of CF in preterm infants should not be delayed with the aim to prevent overweight and obesity.

*Certainty of evidence: Moderate.*

*Grade of recommendation: Strong.*

### Is there a link between early CF and allergy?

The retrospective case-control study by Yrjänä and coll. showed that very early introduction of CF does not affect the incidence of allergy or atopic manifestations among preterm infants, suggesting that their gut-associated lymphoid tissue is ready for CF within 3 to 6 months of chronological age, regardless of GA at birth [49].

Conversely, Morgan et al. showed that preterm infants introduced early (within 17 weeks' CA) to at least four solid foods featured a higher risk of eczema in infancy [50] (Table 5). Despite limited evidence, a recent systematic review suggested that gluten and allergenic foods introduction should not be delayed in preterm infants starting CF. Gluten and allergenic foods should be offered any time after 4 months of CA, irrespective of infants’ relative risk of developing allergy. Limiting the amount of gluten during infancy might be desirable [74].

**Table 5:** Main features of observational studies assessing the relationship between CF introduction in preterm infants and later onset of allergy

Author, Year	Study Design	Sample size	Results
Yrjänä, 2018 [49]	Observational study	Retrospective analysis of data from 464 preterm infants to investigate whether early CF influences the incidence of food allergy or atopic dermatitis among	CF was started at the median CA of 1.4 months for all preterm infants. The incidence either of food allergies or of atopic dermatitis did not differ significantly between preterm infants and controls at 1 and 2 years.
Morgan, 2004 [50]	Observational study	Assessment of CF-related risk factors for eczema at 12 months CA in 257 preterm infants	Introduction of 4 solid foods within 17 weeks CA, male gender, family history of atopy in parents acted as risk factors for the onset of eczema by 12 months CA.

*CA* corrected age, *CF* complementary feeding.

*Recommendations/Statements*

- The introduction of allergenic foods (e.g., eggs, fish, tomato, peanuts) may not be delayed in preterm infants.

*Certainty of evidence: Very Low.*

*Grade of recommendation: Weak.*

### Are vegetarian and vegan weaning regimens feasible in preterm infants?

Vegetarian and vegan diets are increasingly popular [32] also among parents who are reported to ask their paediatricians for alternative weaning regimens with significant frequency [51, 52]. Paediatricians often are not prone to support parents in their decision mainly for the concerns regarding safety of alternative weaning regimens. Indeed, scientific societies [14, 75] encourage weaning regimens based on a large variety of foods and stand against alternative weaning methods due to the risk of nutritional deficiencies and long-term detrimental effects (e.g., failure to thrive, rickets, irreversible cognitive deficits, death). Alarmingly, the sceptical approach of paediatricians jeopardizes the alliance with parents [51, 65] who prefer to adhere to alternative diets with scarce guidance from healthcare professionals, whereas the collaboration between parents, paediatricians and dieticians should be strongly advocated to ensure both a comprehensive growth and development assessment, and an accurate diet planning.

Due to the shortage of consistent data supporting the safety and feasibility of alternative weaning regimens (Table 6), they should be carefully planned for preterm infants, who are a rather delicate population [52]. Parents strongly willing to adhere to alternative weaning regimens should be guided in the process by nutritional experts.

**Table 6:** Main features of studies assessing vegetarian and vegan weaning regimens

Author, Year	Study Design	Sample size	Results
Bivi, 2021 [51]	Observational study	National cross-sectional survey on 176 Italian parents of children following a vegan diet	Nearly 72% of the children enrolled in the study had been on a vegan diet since weaning. Primary care paediatricians were often (70.8%) perceived as sceptical or against a vegan diet. Nearly 70% of the parents relied on medical dietitians, and 28.2% on nutritionists/dietitians for dietary counselling.
Baldassarre, 2020 [52]	Observational study	﻿Survey on 360 Italian families to assess ﻿the prevalence of vegetarian and vegan weaning	﻿Nearly 10% of infants were weaned according to a vegetarian or vegan diet. Almost half of parents perceived their primary care paediatrician as unable to provide sufficient information on unconventional CF and 77.4% of parents reported the paediatrician’s resistance towards alternative CF methods.
Farella, 2020 [65]	Case report	n.a.	Case report on a 22-month-old boy with failure to thrive probably due to an unbalanced vegetarian diet. Difficulty in establishing a therapeutic alliance between parents who follow alternative regimens and the paediatrician is highlighted.

*CF* complementary feeding.

Consumption of foods low in fibre and rich in calcium, iron, zinc, iodine, and DHA together with the supplementation of vitamin D and B12 (in case of vegan diet) are recommended [52]. Infants should also be carefully assessed and monitored for sign and symptoms of nutritional deficits.

*Recommendations/Statements*

- Vegetarian and vegan weaning may be carefully planned in preterm infants.

*Certainty of evidence: Very Low.*

*Grade of recommendation: Weak.*

### Which milk should be consumed during CF?

Similarly to in-hospital nutrition, also at home the main options are human milk (HM), raw or fortified, and formula milk adapted for preterm infants. Despite the fact that fortified HM may help ensure adequate growth [33, 53], the use of fortifiers at home may be troublesome, hence parents should be carefully informed on the importance of continuing fortification after discharge from hospital to improve growth and support breastfeeding, [73] in a period when feeding and sucking competency on the breast are usually improved [53]. Hence, mother’s milk supplementation is often discontinued, exposing the infant to the risk of nutritional deficits and decreased weight gain soon after discharge [34, 53]. Whether exclusive breastfeeding at discharge and suboptimal initial weight gain in preterm infants increase the odds of later cognitive impairment is still matter of debate [1, 53]. The so-called “apparent breastfeeding paradox” clearly describes that very preterm infants started on breast milk early in the course of their life may feature better neurodevelopmental outcomes in spite of suboptimal initial weight gain, thus encouraging the use of breastfeeding in preemies [53].

According to ESPGHAN, exclusive breastfeeding, mixed feeding (in case of insufficient amounts of breast milk) or standard infant formula enriched with LCPUFA should be preferred for infants without EUGR. In contrast, in case of EUGR or high risk of long-term growth failure, infants should be fed up to 40 (but possibly 52) weeks' postmenstrual age with fortified HM or formula milk adapted for preterm infants featuring high protein contents, calcium, phosphorus, zinc, and LCPUFA [67].

*Recommendations*

- Infants without EUGR may be fed with exclusive breastfeeding, mixed feeding (in case of insufficient amounts of breast milk) or standard infant formula enriched with LCPUFA.
- Infants with EUGR or at high risk of long-term growth failure may be fed with fortified HM or formula milk adapted for preterm infants as long as necessary to gain an optimal weight for CA.

*Evidence quality: Low.*

*Grade of recommendation: Weak.*

## Conclusions

To the best of our knowledge, the present position paper by joint societies (SIP, SIN and SIGENP) is the first to draw tailored guidance regarding nutrition and complementary feeding of preterm infants.

We suggest that CF in preterm infants should be started between 5 and 8 months of chronological age, taking also into account the limit of 3 months CA to ensure the acquisition of crucial neurodevelopmental skills which allow the consumption of solid foods. As for type of foods to choose, sequence and speed of introduction, the same guidelines available for term infants should be applied also for ex-preemies. CF should be started encompassing sources of carbohydrates, proteins and vegetable fats (although whether a specific type of vegetable fat should be preferred is still unknown). Attention should be paid to the intake of micronutrients (e.g., iron and vitamins).

A multidisciplinary assessment to evaluate when and how CF should be started is recommended for preterm infants with oral dysfunctions or comorbidities.

According to current knowledge, the timing of CF in preterm infants is unlikely to influence the incidence of overweight and obesity in childhood and adulthood. Thus, it is not necessary to delay the start of CF in preterm infants to prevent overweight and obesity. Similarly, the introduction of allergenic foods (e.g., eggs, fish, tomato, peanuts) should not be delayed in preterm infants.

Vegetarian and vegan weaning should be carefully planned in preterm infants, in order to prevent detrimental effects due to nutritional deficiencies.

Future research should also aim at providing tailored recommendations for subgroups of preterm infants, such as late preterm neonates, that feature higher risk of lower weight and height during childhood, insulin resistance, glucose intolerance, and high blood pressure compared to term neonates, and whose nutritional requirements are still matter of debate [82].

## Data Availability

All relevant data are included in the article.

## Abbreviations

AGA: Appropriate for gestational age
BMI: Body mass index
BW: Birth weight
CA: Corrected age
CF: Complementary feeding
DHA: Docosahexaenoic acid
EFSA: European Food Safety Authority
ELBW: Extremely low birth weight
ELGAN: Extremely low gestational age neonates
EUGR: Extrauterine growth retardation
GA: Gestational age
GRADE: Grading of Recommendations, Assessment, Development and Evaluation
HM: Human milk
IGF-1: Insulin-like growth factor-1
IUGR: Intrauterine growth restriction
LARNs: Reference intake Levels of Nutrients
LCPUFA: Long-chain polyunsaturated fatty acids
LGA: Large for gestational age
OFP: Oral feeding protocol
PA: Postnatal age
PRISMA: Preferred Reporting Items for Systematic Review and Meta-Analysis
PWS: Preterm weaning strategy
RCT: Randomized controlled trial
SGA: Small for gestational age
SIGENP: Italian Society of Paediatric Gastroenterology, Hepatology and Nutrition
SIN: Italian Society of Neonatology
SIP: Italian Society of Paediatrics
